# Ultrasonic features of papillary thyroid microcarcinoma and non-microcarcinoma

**DOI:** 10.3892/etm.2014.1910

**Published:** 2014-08-18

**Authors:** XIAO-LI ZHANG, LIN-XUE QIAN

**Affiliations:** Department of Ultrasound, Beijing Friendship Hospital Affiliated to Capital Medical University, Beijing 100050, P.R. China

**Keywords:** thyroid cancer, papillary, papillary thyroid microcarcinoma, ultrasonography

## Abstract

The present study analyzed the ultrasonic features of papillary thyroid microcarcinoma (PTMC) and papillary thyroid non-microcarcinoma (non-PTMC) with the aim of improving the diagnostic value of ultrasonography. The ultrasonic features of 328 patients with papillary thyroid carcinoma (PTC), as confirmed by pathology, were retrospectively analyzed. Patients were diagnosed with PTMC if the tumor size was ≤10 mm in diameter or non-PTMC if the tumor size was >10 mm in diameter. The shape, ratio of length/width, boundary, echo, peripheral halo ring, calcification, cystic changes, blood flow, lymph node metastasis and additional accompanying thyroid diseases were compared between the patients with PTMC and non-PTMC. In total, 389 nodules were identified in the 328 patients, including 209 PTMC nodules and 180 non-PTMC nodules. The multifocality rate was 15.9% (52/328). Ultrasound scans detected a total of 371 nodules, while 18 PMTC nodules were missed diagnosed and 57 nodules were misdiagnosed as benign nodules. Statistically significant differences in the boundary, ratio of length/width, cystic changes, blood flow and lymph node metastasis were observed between the PTMC and non-PTMC groups (P<0.05). However, no statistically significant differences were observed with regard to the shape, calcification, peripheral halo rings and additional accompanying thyroid diseases between the two groups (P>0.05). Therefore, ultrasound plays an important role in the diagnosis of PTC, with PTMC and non-PTMC exhibiting different ultrasonic performances.

## Introduction

Papillary thyroid carcinoma (PTC) is the most common type of primary malignant thyroid cancer, with the incidence rate increasing in the last decade (1). Due to the insidious onset and slow development of PTC (2), an early diagnosis is important to select the correct treatment strategy and improve prognosis (3). However, in papillary thyroid microcarcinoma (PTMC), the rates of incorrect and missed diagnoses are high due to the atypical clinical symptoms and more aggressive behavior with regional and distant metastases (4,5). Computed tomography, magnetic resonance imaging and isotope examination are all ineffective; therefore, ultrasound examination is the best method of diagnosing PTC (6). However, whether PTMC and papillary thyroid non-microcarcinoma (non-PTMC) exhibit the same ultrasonic performances is controversial (2,7). The aim of the present study was to retrospectively compare the sonographic features of PTMC and non-PTMC, in order to improve the diagnostic value of ultrasonography.

## Materials and methods

### General data

For retrospective analysis, data from a total of 328 patients with PTC, who had undergone thyroid surgery at the Beijing Friendship Hospital (Beijing, China), were collected between June 2010 and October 2013. Of the cases analyzed, 72 were male and 256 were female, aged between 19 and 83 years, with a mean age of 43.6±12.5 years. The case histories ranged between one day and five years. All the patients underwent preoperative ultrasonography within two weeks and a diagnosis of PTC was confirmed by surgery and pathological examination. The study was conducted in accordance with the Declaration of Helsinki and with approval from the Ethics Committee of the Capital Medical University (Beijing, China). Written informed consent was obtained from all the participants.

### Ultrasound examination

Philips iU22 (Philips Ultrasound, Inc., Bothell, WA, USA), GE LOGIQ E9 (GE Healthcare, Wauwatosa, WI, USA) and HI VISION Preirus (Hitachi Medical Corporation, Tokyo, Japan) color ultrasound diagnostic apparatus were used for analysis, with the probe frequency set at 5–12 MHz. The patients were placed in a supine position, exposing the anterior thyroid area, scanning multi-slice display thyroid nodules, number, size, shape, ratio of length/width, boundary, echo, peripheral halo ring, calcification rate, cystic changes, blood flow and accompanying diseases were observed. A PTMC diagnosis was confirmed if the tumor diameter was ≤10 mm, while non-PTMC cases were confirmed with a tumor diameter of >10 mm, according to the World Health Organization criteria (8). The calcification types were as follows: Microcalcification, ≤2 mm in diameter with punctate hyperechoic foci, with or without shadow and a scattered or clustered distribution; and coarse calcifications, >2 mm in diameter with sheet or shell-like hyperechoic foci and shadow. Adler flow grading (9) was determined as follows: Grade 0, no blood flow; grade I, a small amount of blood with 1–2 punctuate or rod-like blood vessels; grade II, medium flow with three or four blood vessels, one of which being longer than the radius of the nodule; grade III, rich in blood and more than four visible blood vessels or interconnected angiogenesis, interwoven into a network (9).

### Statistical analysis

Data were analyzed using SPSS 17.0 software (SPSS, Inc., Chicago, IL USA), and measurement data are expressed as the mean ± standard deviation. Enumeration data were analyzed with the χ^2^ test, while the Wilcoxon rank-sum test was used to analyze the differences in blood flow between the two groups. P<0.05 was considered to indicate a statistically significant difference.

## Results

### Pathological observations

A total of 389 nodules were detected by ultrasound in the 328 cases. Of these, 167 nodules were located in the left lobe, 195 were identified in the right lobe and 27 were present in the isthmus. There were 209 PTMC nodules and 180 non-PTMC nodules. Of the patients examined, there were 52 (52/328, 15.9%) multifocal cases where between two and six nodules were identified, of which 37 were cases of PTMC and non-PTMC combined, while the remaining 15 cases were all PTMC. In total, 58.61% (228/389) of the nodules were found in patients with other thyroid diseases, including Hashimoto’s thyroiditis (HT; 38.60%; 88/228), nodular goiter (52.19%; 119/228), adenoma (1.32%; 3/228) and nodular goiter accompanied with HT or adenoma (7.89%; 18/228); however, statistically significant differences were not observed when comparing the PTMC and non-PTMC cases (P>0.05; Table I). There were 57 PTMC (57/209, 27.27%) and 103 non-PTMC (103/180, 57.22%) cases of lymph node metastasis; the difference between the two groups was statistically significant (P<0.05).

### Ultrasound observations

**Figure 1:**
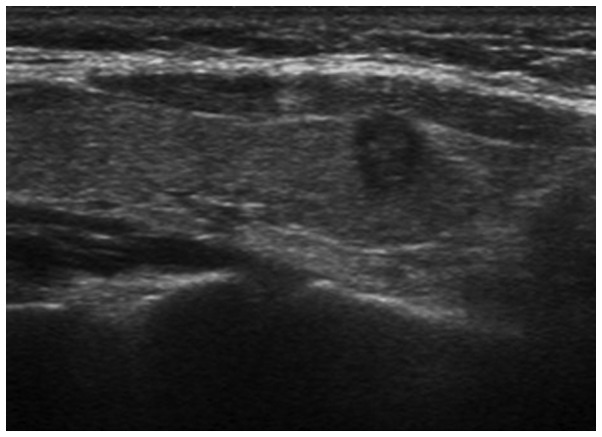
Ultrasound scan of a papillary thyroid microcarcinoma (PTMC) in a 50-year-old female. A hypoechoic nodule, 0.52×0.65 cm in size, was observed in the right lobe. The PTMC has an unclear boundary, an irregular shape and a ratio of length/width of ≥1.

**Figure 2:**
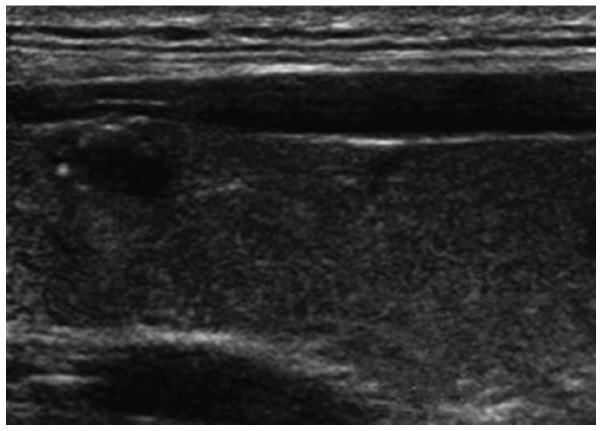
Ultrasound scan of a papillary thyroid microcarcinoma (PTMC) in a 47-year-old female. A hypoechoic nodule, 0.72×0.53 cm in size, was observed in the right lobe. The PTMC has an unclear boundary with microcalcification surrounding the nodule.

**Figure 3:**
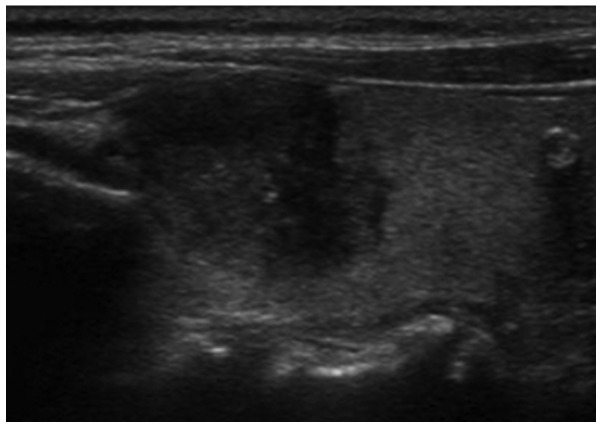
Ultrasound scan of a papillary thyroid non-microcarcinoma (non-PTMC) in a 56-year-old female. A hypoechoic nodule, 2.04×1.76 cm in size, was observed in the right lobe. The non-PTMC has a less clear boundary, shallow lobulation and microcalcification.

**Figure 4:**
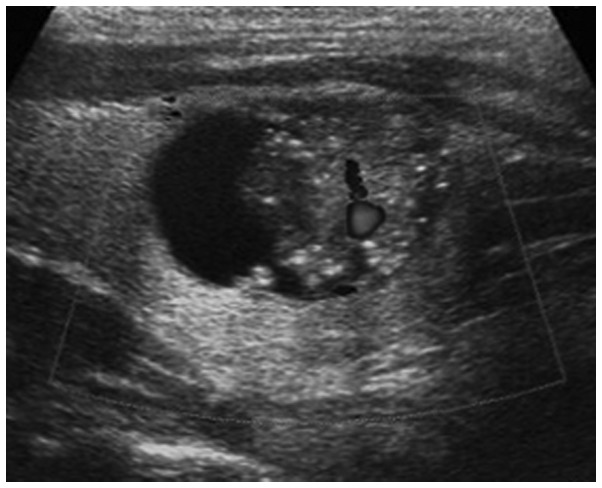
Ultrasound scan of a papillary thyroid non-microcarcinoma (non-PTMC) in a 41-year-old male. A cystic-solid nodule, 2.36×1.65 cm in size, was observed in the left lobe. The non-PTMC has an unclear boundary of the solid component, an irregular shape, dense microcalcification and strip-like blood vessels.

Ultrasound scans detected 371 nodules, ranging in size between 0.24×0.32 and 5.35×3.86 cm. Of these, 191 were PTMC nodules and 180 were non-PTMC nodules (Figs. 1–4). The scans missed 18 nodules, which were all PTMCs with a diameter of ≤3 mm. A total of 57 nodules were misdiagnosed, including 24 PTMCs and 33 non-PTMCs, of which 52 were misdiagnosed as nodular goiter and five were misdiagnosed as adenomas.

With regard to the PTMC nodules, an unclear boundary and length/width ratio of ≥1 accounted for 87.96% (168/191) and 46.60% (89/191) of cases, respectively. By contrast, in the non-PTMC nodules, an unclear boundary and length/width ratio of ≥1 accounted for 69.44% (125/180) and 3.89% (7/180) of cases, respectively. The blood flow in the PTMC nodules was predominantly grade 0-I, accounting for 81.68% (156/191) of the nodules. In the non-PTMC nodules, the blood flow was mainly grade II-III, accounting for 70.00% of the nodules (126/180; Table II).

There were no cystic changes observed in the PTMC nodules; however, the rate of cystic change in the non-PTMC cases was ~16.67% (30/180), and the difference between the two carcinoma types was statistically significant (P<0.05). A total of 63.33% (19/30) of the non-PTMC cases with cystic changes had microcalcifications in the solid component. PTMC nodules were mainly hypoechoic (167/191; 87.43%); however, the echogenicity of the non-PTMC nodules varied, with 53.89% (97/180) of the nodules being hypoechoic. The difference in echogenicity between the two carcinoma types was statistically significant (P<0.05). Microcalcifications and coarse calcifications were present in 37.70% (72/191) and 18.32% (35/191) of the PTMC nodules, respectively, and 42.78% (77/180) and 18.33% (33/180) of the non-PTMC nodules, respectively. No statistically significant differences in the calcification rate and ratio were observed between the two groups (P>0.05). Furthermore, the two groups were rarely accompanied by a peripheral halo ring (Table III).

## Discussion

PTC is the most common type of thyroid cancer, with a low degree of malignancy and a good prognosis. Thyroid microcarcinoma refers to nodules with a diameter of ≤10 mm, with or without regional or distant lymph node metastasis. The incidence rate of PTMC is 2.0–35.6% in autopsy (10). PTMC cases accounted for 53.73% (209/389) of the total nodules analyzed in the present study. PTMC presents as isolated nodules that coexist with a larger tumor or exist in large benign nodules (11). In the current study, 18 cases of PTMC were missed by ultrasound scans as they were identified as local carcinogenesis in larger nodules. There is no pathological difference between PTMC and non-PTMC. At present, more research has focused on the molecular level (12,13). The growth of PTMC nodules is slow; a previous study revealed that the 30-year recurrence rate was 40% in patients of >55 years-old, and the prognosis rate was better in younger individuals (14).

Multiple nodules are one of the clinical features of PTC, with a reported occurrence rate of 18–87% (15). The occurrence rate was slightly lower in the present study, possibly due to a smaller range of pathological specimens. Pathological examination of the entire gland may result in a higher incidence due to the fact that contralateral disease is also detected (15). A previous study observed that there was a high degree of malignancy in multiple nodules, as well as cervical lymph node metastasis and a larger proportion of thyroid infiltration; however, no statistically significant differences were observed in the disease-specific and total mortality rates when comparing PTMC and non-PTMC patients (16). Whether the formation of multiple foci occurs from the gland or is of polyclonal origin remains controversial. A previous study (17) confirmed that a considerable part of multifocal PTC is of polyclonal origin. Thus, there are limits to detecting multifocal nodules by ultrasound scans as they may merge with other benign thyroid lesions.

PTMC nodules are primarily hypoechoic, which may be associated with the low degree of differentiation in cancer cells, fewer interstitial components and a good sound transmission in the tumor. With nodule growth, blood vessels and fibrous tissue undergo hyperplasia, causing the echogenicity to vary. As the tumors grow faster, liquefaction necrosis and cystic changes occur (18). In the present study, cystic changes were observed in non-PTMC, with more than half of the cases accompanied by microcalcifications within the solid component. A previous study demonstrated that microcalcifications in the solid component of cystic-solid nodules have a higher specificity for the diagnosis of PTC (19).

Microcalcifications may reflect psammoma bodies, which are the most specific diagnostic indicators of PTC (20). A previous study (21) revealed that microcalcifications occur as a result of good growth of autocrine tumor cells, while coarse calcification occurs as a result of the rapid growth of cancer cells, tissue hyperplasia, degeneration and then calcium deposition, known as dystrophic calcification. In the current study, microcalcifications accounted for 40.16% (149/371) of cases, while coarse calcifications were present in 18.33% (68/371) of cases and no calcification was observed in 41.51% (154/371) of the total nodules detected. No statistically significant difference in calcification was identified between the groups.

Neovascularization provides nutrients for the growth of malignant tumors. In the present study, the blood flow of the PTMC nodules was primarily grade 0-I, whereas the blood flow in the non-PTMC nodules was mainly grade II-III. This difference may be due to the fact that there is no neovascularization in non-PTMC or because low-velocity blood signals were not shown due to improper adjustment of the instrument.

PTC often coexists with nodular goiter, HT and adenoma. In the present study, the detection rate of PTC was significantly lower when the disease was accompanied with nodular goiter, as atypical nodules may be ignored when scanning multiple foci. However, the most important reason for misdiagnosis is that the sonographer may lack knowledge of the local carcinogenesis of benign nodules. The pathological basis of HT is lymphocytic infiltration and follicular cell destruction. A previous study revealed that PTC was accompanied by HT in ~18% of cases (22). HT may destroy follicular cells and reduce the secretion of thyroid hormones. In addition, feedback causes increased secretion of thyroid-stimulating hormone, which stimulates hyperplasia of the follicular epithelium. From investigations at a molecular level, including investigations into rearranged during transfection protooncogene/PTC and cytokeratin-19, certain studies have hypothesized that HT is a precancerous form of PTC (23,24). Thus, nodules in HT should be closely followed-up.

In conclusion, ultrasound has an important value in the diagnosis of PTC. Specific differences exist in the ultrasonic features between PTMC and non-PTMC; however, the diagnosis of benign nodules accompanied by the occasional microcancer has limitations. For cases which are difficult to diagnose, fine-needle aspiration or ultrasound-guided biopsy should be considered.

## Figures and Tables

**Table I:** Comparison of additional accompanying thyroid diseases in the patients.

Group	HT	Nodular goiter	Adenoma	Nodular goiter with HT	Nodular goiter with adenoma
PTMC, n	50	57	1	7	3
Non-PTMC, n	38	62	2	6	2
Total, n	88	119	3	13	5

PTMC, papillary thyroid microcarcinoma; non-PTMC, papillary thyroid non-microcarcinoma; HT, Hashimoto’s thyroiditis.

**Table II:** Comparisons of the shape, boundary, ratio of length/width and blood flow.

	Shape		Boundary		Ratio of length/width		Blood flow^b^			
				
Group	Regular	Irregular	Clear	Unclear	<1	≥1	0	I	II	III
PTMC, n	31	160	23	168	102	89	52	104	24	11
Non-PTMC, n	26	154	55	125	173	7	9	45	84	42
χ^2^-value	0.23		19.13		88.12		−6.76^a^			
P-value	0.63		<0.01		<0.01		<0.01			

a Z-value.

b Assessed by Adler flow grading.

PTMC, papillary thyroid microcarcinoma; non-PTMC, papillary thyroid non-microcarcinoma.

**Table III:** Comparisons of the cystic changes, echo of solid component, peripheral halo rings and calcification type.

	Cystic changes		Echo of solid component		Peripheral halo ring		Calcification		
				
Group	Solid	Cyst-solid	Hypoechoic	Equal/hyperechoic	With	Without	Without	Micro-	Coarse
PTMC, n	191	0	167	248	12	179	84	72	35
Non-PTMC, n	150	30	97	83	11	169	70	77	33
χ^2^-value	34.63		50.81		0.01		1.17		
P-value	<0.01		<0.01		0.905		0.56		

PTMC, papillary thyroid microcarcinoma; non-PTMC, papillary thyroid non-microcarcinoma.
